# Fused Deposition Modeling as a Possible Approach for the Preparation of Orodispersible Tablets

**DOI:** 10.3390/ph15010069

**Published:** 2022-01-05

**Authors:** Thao Tranová, Jolanta Pyteraf, Mateusz Kurek, Witold Jamróz, Witold Brniak, Dita Spálovská, Jan Loskot, Karolina Jurkiewicz, Joanna Grelska, Daniel Kramarczyk, Jitka Mužíková, Marian Paluch, Renata Jachowicz

**Affiliations:** 1 Department of Pharmaceutical Technology, Faculty of Pharmacy in Hradec Králové, Charles University, Akademika Heyrovského 1203, 500 05 Hradec Králové, Czech Republic; tranthip@faf.cuni.cz (T.T.); muzikova@faf.cuni.cz (J.M.); 2 Department of Pharmaceutical Technology and Biopharmaceutics, Jagiellonian University Medical College, Medyczna 9, 30-688 Krakow, Poland; mateusz.kurek@uj.edu.pl (M.K.); witold.jamroz@uj.edu.pl (W.J.); w.brniak@uj.edu.pl (W.B.); renata.jachowicz@uj.edu.pl (R.J.); 3 Department of Analytical Chemistry, University of Chemistry and Technology Prague, Technická 5, 166 28 Prague, Czech Republic; dita.spalovska@vscht.cz; 4 Zentiva, k.s, U Kabelovny 130, 102 37 Prague, Czech Republic; 5 Department of Physics, Faculty of Science, University of Hradec Králové, Rokitanského 62, 500 03 Hradec Králové, Czech Republic; jan.loskot@uhk.cz; 6 A. Chełkowski Institute of Physics, University of Silesia in Katowice, ul. 75 Pułku Piechoty 1, 41-500 Chorzów, Poland; karolina.jurkiewicz@us.edu.pl (K.J.); joanna.grelska@us.edu.pl (J.G.); daniel.kramarczyk@smcebi.edu.pl (D.K.); marian.paluch@us.edu.pl (M.P.)

**Keywords:** fused deposition modeling, 3D printing, hot-melt extrusion, solid dosage forms, orodispersible tablets, paracetamol, domperidone, disintegration time

## Abstract

Additive manufacturing technologies are considered as a potential way to support individualized pharmacotherapy due to the possibility of the production of small batches of customized tablets characterized by complex structures. We designed five different shapes and analyzed the effect of the surface/mass ratio, the influence of excipients, and storage conditions on the disintegration time of tablets printed using the fused deposition modeling method. As model pharmaceutical active ingredients (APIs), we used paracetamol and domperidone, characterized by different thermal properties, classified into the various Biopharmaceutical Classification System groups. We found that the high surface/mass ratio of the designed tablet shapes together with the addition of mannitol and controlled humidity storage conditions turned out to be crucial for fast tablet’s disintegration. As a result, mean disintegration time was reduced from 5 min 46 s to 2 min 22 s, and from 11 min 43 s to 2 min 25 s for paracetamol- and domperidone-loaded tablets, respectively, fulfilling the European Pharmacopeia requirement for orodispersible tablets (ODTs). The tablet’s immediate release characteristics were confirmed during the dissolution study: over 80% of APIs were released from printlets within 15 min. Thus, this study proved the possibility of using fused deposition modeling for the preparation of ODTs.

## 1. Introduction

Orodispersible tablets (ODTs) are recognized as a dosage form that plays a significant role in increasing patient compliance. ODTs can be easily administered to patients with swallowing problems, i.e., with oropharyngeal dysphagia, as well as being suitable for children and patients with nausea [1,2,3,4]. Furthermore, it has been proven that orodispersible tablets increase the compliance of patients with depression who are often compelled to life-long pharmacotherapy [5].

European Pharmacopeia (Ph. Eur., 10th Edition) defines orodispersible tablets as tablets that disperse inside the mouth readily before swallowing [6]. According to the monograph included in Ph. Eur., ODTs should disintegrate within 3 min during the disintegration test. However, United States Pharmacopeia (USP) refers to the *Guidance for Industry: Orally Disintegrating Tablets* issued by the Food and Drug Administration (FDA)*,* which demands disintegration time below 30 s [7]. Other principal characteristics of ODTs may include the faster onset of action, increased bioavailability of some active pharmaceutical ingredients (APIs), and accuracy in a dose, which in comparison to liquids leads to a higher level of safety of the treatment [8].

Examples of preparation methods for ODTs are direct compression, heat molding, lyophilization, cotton candy method [9], and 3D printing. The latter method had been undergoing a revolution in the pharmaceutical field since the first US Food and Drug Administration (FDA) approval of the 3D printed medicine, Spritam^®^, orodispersible tablets used for epilepsy treatment [10]. Unlike the other methods of manufacturing solid dosage forms, 3D printing technologies allow the production of small batches of customized, very complex structures and are considered as a potential way to support individualized pharmacotherapy in hospitals and community pharmacies [11,12]. Such a solution could be particularly advantageous for pediatric and geriatric populations, which often demand different dosage adjustments. Moreover, it is suitable for APIs with a narrow therapeutic index enabling the fabrication of a dosage form containing a precise drug dose [13].

Because of the great potential of the 3D printing method in the production of personalized dosage forms, Rautamo et al. ascertained the perception of healthcare professionals on the topic of 3D printing in hospitals. They concluded that the important features of 3D printed dosage forms include rapid disintegration after oral administration, which is particularly suitable for children, as well as short on-demand delivery and easy identification [14]. This can be solved by printing orally disintegrating tablets.

Fused deposition modeling (FDM) is one of the most widely described low-cost and rapid ways to prepare 3D printed (3DP) tablets. It enables quick and simple adjustment of printing parameters and provides products with good mechanical properties [15]. Moreover, due to the prior preparation of the filaments by hot-melt extrusion and conditions during the printing process, it is possible to obtain solid amorphous dispersion of an API in a polymer matrix, and thus increase the dissolution rate [16]. Although many publications indicate the possibility of FDM printing of immediate-release tablets, including fast-disintegrating dosage forms, to our knowledge, only one article refers to the manufacturing of tablets with a disintegration time shorter than 3 min, i.e., meeting Ph. Eur. requirements for ODTs [17].

FDM-printed immediate-release tablets are made of water-soluble filament-forming polymers such as poly(vinyl alcohol) [18], PEG-PVA copolymer (Kollicoat IR) [17,19], hydroxypropyl-cellulose SSL [20], copovidone (Kollidon VA64) and povidone (Kollidon 12PF) [21]. Sometimes additional polymers, plasticizers, or disintegrants are added to formulations to accelerate the disintegration of the dosage form and dissolution of API. In our previous work, we analyzed the effectiveness of a 4% addition of superdisintegrants: sodium starch glycolate, crospovidone, and croscarmellose sodium on the ketoprofen dissolution rate from 3DP tablets. The fastest release was observed for formulations containing crospovidone [22]. The impact of disintegrants has also been analyzed by several other research groups [23,24,25]. Hussain et al. analyzed HPC SL-based formulations containing captopril and disintegrants. The addition of sodium starch glycolate and sodium croscarmellose has improved the in-vitro drug release and tablets’ disintegration time when used in a 10% concentration [23]. 

Another possibility to accelerate the tablet disintegration and drug dissolution is to change the structure of the tablet, in particular, to increase the surface area of the tablet, e.g., by printing tablets with low infill density [26,27] or by preparation of channeled tablets [28,29]. Sadia et al. investigated oblong tablets with channels of different diameters arranged along or across the tablet. The complex design of these tablets not only increased the surface area but also facilitated media penetration through the structure [28]. Moreover, Arafat et al. developed tablets containing built-in gaps that accelerated drug release. The result was tablets that met the USP criteria for immediate-release products without the use of disintegrants [29].

Regardless of the excipients used and structure modifications applied, the properties of the API, especially in high drug-loaded formulations, have a significant effect on the printability and properties of the dosage form. For this reason, the development of the shape and composition of universal, fast disintegrating formulations, regardless of the properties of the API, is challenging.

Our work aimed to develop orodispersible tablets using the FDM 3D printing method and analyze their properties. We evaluated the effect of tablets’ spatial structure, the addition of plasticizers, and storage conditions on the tablets’ disintegration time and drug dissolution rate. We analyzed tablets containing two APIs: paracetamol or domperidone, classified into different groups according to the Biopharmaceutical Classification System (BCS), and characterized by different thermal properties. As a result, we have developed formulations containing APIs with different properties, with a disintegration time of less than 3 min, meeting the Ph. Eur. requirements for ODTs.

## 2. Results and Discussion

Paracetamol and domperidone were selected as model drugs with different properties. Paracetamol is an analgesic and antipyretic agent with a dosage ranging from 500 mg to 1000 mg. In addition, it is used for infants and children in a dose in the range of 10–15 mg/kg BW [30]. It is classified as a BCS class I compound since it is highly soluble and permeable [31]. Paracetamol is slightly soluble in water [32]. Its melting temperature was determined by differential scanning calorimetry (DSC) as 168 °C. We used paracetamol in the granulate form because of the feeding issues during the hot-melt extrusion process, resulting from the poor flowability of the API and its high content in the formulations, i.e., 40%. Domperidone is used in the treatment of nausea and vomiting in the 10 mg strength. It is practically insoluble in water [33]. Domperidone is classified as class II according to the BCS classification system, which means that its bioavailability is limited by poor solubility [34]. Its high melting temperature, i.e., 247 °C makes it especially hard to process with hot-melt extrusion and fused deposition modeling technologies. In all experiments, it was used as a pure API powder as it was used in smaller concentrations in the filament formulations and its properties did not affect the final blend flowability as much as in the case of paracetamol. Parteck^®^ MXP poly(vinyl alcohol) was used as a filament-forming polymer with wide processing temperatures and high thermal stability (degradation temperature above 250 °C). The polymer has semicrystalline nature with a low glass transition temperature of 45 °C and a melting temperature of 170 °C. It has been proven that it can be processed with a wide range of APIs even with high melting temperatures [35]. The thermograms of raw materials are presented in the Supplementary Materials. We used crospovidone (PVP CL, Kollidon^®^ CL) as a superdisintegrant in all formulations as we have previously proven its efficiency in 3D printing technology [22].

### 2.1. Processability of HME, Drug Content, and Texture Analysis of Filaments

As a result of the hot-melt extrusion processes, we obtained four drug-loaded filaments, two for each API. Initially, we produced filaments containing the 40% of paracetamol or 10% of domperidone, 5% of crospovidone and polyvinyl alcohol (PVA), encoded as PAR and DOM for paracetamol- and domperidone-loaded formulations, respectively. Next, to modify the properties of the tablets, we extruded filaments containing 10% mannitol (reducing the amount of PVA), encoded as PAR + M and DOM + M, depending on the API used. Additionally, the DOM + M filament contained a higher amount of domperidone compared to the DOM. The detailed compositions of individual formulations are presented in Table 1. 

The hot-melt extrusion processes were optimized to achieve a uniform filament diameter of around 1.75 mm. The extrusion was smooth and robust for paracetamol formulations despite the 40% drug loading.

The achieved paracetamol-loaded filaments had dimensions close to the established diameter and its uniformity was also at an acceptable level. The mechanical strength evaluated in the stretching test, presented in Table 2, showed that filaments were characterized by high mechanical resilience with the tensile strength reaching up to 15.34 MPa and 10.66 MPa for PAR and PAR + M filaments, respectively. The calculated Young modulus values indicate that the elasticity and stiffness of the filaments were good enough for the 3D printing process. We have shown in our previous work that the filaments with Young modulus values ranging from ca. 400 MPa up to more than 2100 MPa are printable in our set-up [22]. The DOM + M filament was 2-times more elastic in comparison to the filament without mannitol. It was caused by the plasticizing effect of the mannitol. The effect was not pronounced in the case of paracetamol filaments due to the simultaneous increase of paracetamol’s crystallinity in the PAR + M filament, which lowered the elasticity.

The temperature profiles have to be adjusted in order to make the extrusion possible, especially for domperidone, which is characterized by high melting temperature. In this case, a high torque, reaching 80% of the maximum extruder torque, was produced during the process even with a small output. To achieve the higher domperidone loading and to improve extrudability, mannitol was introduced as a plasticizer. It resulted in a reduced torque of around 50% of maximum and the possibility to achieve 20% of drug loading. The obtained filaments had a higher diameter than expected which resulted from post extrusion swelling of the material (Barus effect). The tensile strength and Young modulus of the domperidone-loaded filaments were higher in comparison with paracetamol-loaded filaments because of lower drug-loading and higher PVA content in the filament. The mechanical properties of all tested filaments were good and indicated that the filaments were printable.

**Figure 1:**
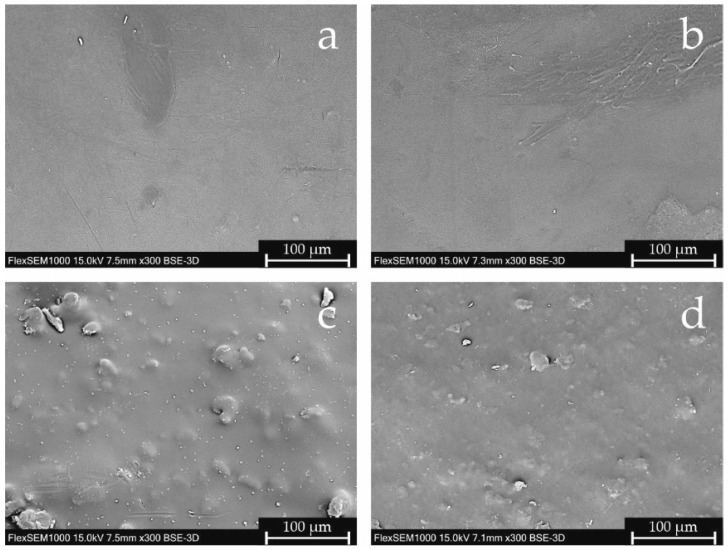
SEM pictures of filaments’ surfaces: (**a**) PAR, (**b**) PAR + M, (**c**) DOM, (**d**) DOM + M (magnification 300×).

The extruded paracetamol filaments were smooth, while domperidone extrudates had a rough surface. It was confirmed by scanning electron microscopy (SEM) pictures analysis. In Figure 1a,b the smooth surface of the paracetamol filaments is presented; in contrast, in pictures c and d, the presence of sharp-edged particles on the surfaces of both domperidone-loaded filaments can clearly be seen. Interestingly, the particles observed on the surface of the DOM filament are better marked and bigger, which may result from different extrusion conditions. Formulation DOM + M was extruded at a higher barrel but lower die temperature. Thus, the crystals of domperidone in the DOM + M extrudate have been partially melted and were better dispersed in the polymer matrix.

### 2.2. 3D Printing Proces

All obtained filaments, regardless of their composition, were printable. However, due to the small printer’s nozzle diameter (equal to 0.2 mm) and the significant differences between the properties of the APIs used, the development of the appropriate printing settings for each formulation was challenging. While in the case of most of the filaments we had tested so far, a slight change in the printing temperature did not significantly affect the quality of the tablets, however, in this case, it turned out to be the most important parameter. The use of too low a printing temperature caused a blockage of the printer nozzle and, as a result, breaks in the applied paths. On the other hand, raising the temperature caused too much plasticity in the extruded mass, which formed arcs and stuck to the previously printed layers of tablets. Optimal printing temperatures were 183 °C and 250 °C for PAR and DOM formulations, respectively. Under applied temperature conditions, the material extruded through the nozzle was heated enough not to block the printer nozzle and cooled rapidly after deposition to reproduce the porous structures of the designed tablets.

The addition of mannitol caused significant changes in the properties of the filaments, which made it necessary to lower the printing temperatures. In the case of PAR + M formulation, the printing temperature was reduced to 165 °C, while for DOM + M composition, printable at 220 °C, this difference is even greater compared to the formulation without plasticizer, even though the filament contained two times more domperidone. For all formulations, it was possible to print tablets containing the assumed APIs’ doses of 100 mg or 10 mg for tablets containing paracetamol and domperidone, respectively.

**Figure 2:**
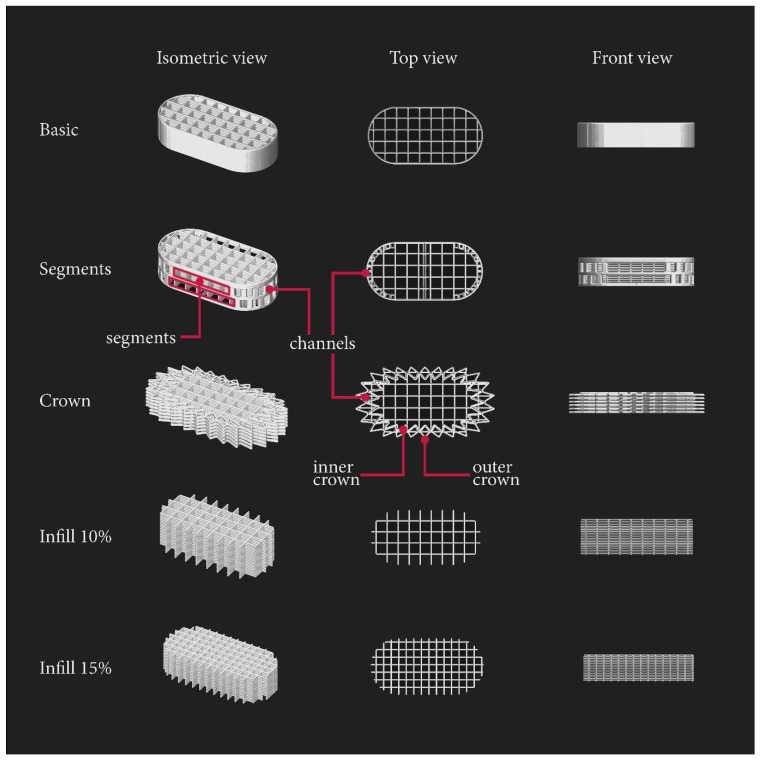
Designed tablets’ shapes from isometric, top, and front view.

To evaluate the influence of tablets’ surface area on disintegration time, we used PAR and DOM filaments to print formulations with five different shapes, presented in Figure 2:

- Basic—tablet consisting of one outline and rectilinear infill with a density of 10%;
- Segments—modified basic shape containing outline holes;
- Crown—tablet based on two-layers parts of 10% rectilinear infill surrounded by crown-shaped rings, with two different sizes, printed alternately: the first two layers bigger (outer crown), then smaller (inner crown), etc.;
- Infill 10%—porous structure without any outline, consisting only of 10% rectilinear infill;
- Infill 15%—porous structure without any outline, consisting only of 15% rectilinear infill.

Moreover, tablets characterized by the highest surface area/mass ratio, i.e., crown, 10% and 15% infill were also printed tablets using filaments containing the addition of mannitol (PAR + M and DOM + M, respectively).

### 2.3. Disintegration

#### 2.3.1. The Effect of Storage Conditions

Based on the results reported by Wei et al., PVA has a hygroscopic nature in physical mixtures, melt extrudates, and eventually in printed tablets at relative humidity (RH) up to 60% [36]. Since the content of our formulations is predominantly made of PVA (Table 1), the basic-shape tablets made of PAR and DOM filaments were printed and evaluated on their disintegration time to understand the impact of different storage conditions on the tablets’ disintegration. Tablets were weighed immediately after printing on an analytical balance and stored in a climate chamber (25 °C, 60% RH), in the room conditions, and in a desiccator for 24 h. After storage, the tablets were reweighed with the purpose of weight change observation. The tablets stored in the climate chamber absorbed the most moisture compared to those stored under room conditions or in the desiccator (Table 3). The DOM-based tablets stored at room conditions as well as climate chamber gained a greater amount of weight in comparison to tablets made of PAR filament, probably due to the highest amount of hygroscopic PVA (Table 1). For tablets stored in the desiccator, a slight weight loss was observed due to the evaporation of water.

The disintegration time test revealed that in the case of tablets containing paracetamol, storage in the desiccator appeared to be the most beneficial since the disintegration time was the shortest (4 min 28 s ± 7 s). Presumably, the tablet structures stored in the climate chamber or at room conditions absorbed moisture which resulted in forming a hydrogel of PVA in the printed paths of the tablet. These paths soaked with moisture stuck together, forming a structure that did not allow the disintegrating medium to penetrate through. In the case of tablets stored in the desiccator, the individually printed paths did not stick together since no moisture was absorbed, and therefore the water penetration and tablet disintegration were faster.

The shortest disintegration time of tablets made of the DOM filament was found when stored in the climate chamber. However, the standard deviation was high (8 min 46 s ± 1 min 49 s), indicating that these tablets’ disintegration times are comparable to the disintegration times of tablets stored in the desiccator (9 min 54 s ± 17 s). Therefore, we decided to store all the subsequently printed tablets in the desiccator.

#### 2.3.2. Surface Area Calculations and Correlation between Surface Areas and Disintegration Time

In several studies, it has been proven that the surface area/volume ratio has a significant effect on the dissolution rate of API from the 3D printed tablets [28,37,38]. Tablets disintegration can be considered as a prior stage before the dissolution [39], and since the disintegration assessment was more important for our study, we concentrated on the design of different tablets shapes, calculated the surface and surface/mass ratio presented in Table 4, and measured the disintegration time. All tablets were stored in a desiccator for 24 h prior to the testing.

The basic shape, which consisted of one outline and 10% rectilinear infill, was considered as the fundamental shape upon which we based the design of our other prototypes. During the disintegration of the basic-shaped tablet, the infill initially disintegrated within an estimated 40 s. However, the remaining outline formed a long glued and swollen mass which took a long time to break into smaller fragments that passed through the mesh of the disintegration apparatus (disintegration times for PAR_basic and DOM_basic were 4 min 28 s ± 7 s and 9 min 54 s ± 17 s, respectively). The reason for this was that the printed layers of the outline touched each other creating a non-permeable barrier (Figure 2: basic; front view).

**Figure 3:**
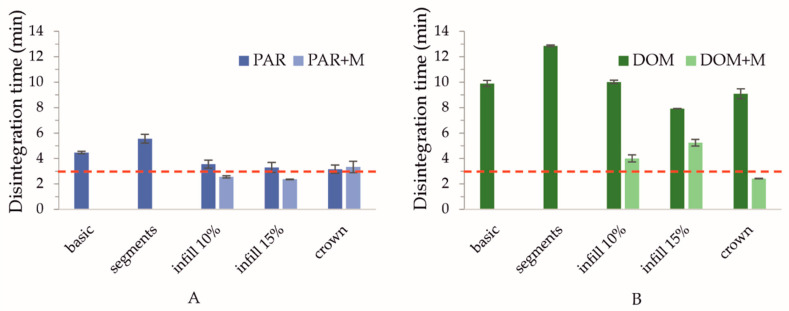
Disintegration time of paracetamol- (**A**) and domperidone-loaded tablets (**B**) stored for 24 h in a desiccator (22.7 °C, 35% RH). The red dashed line represents the maximum disintegration time of the ODTs according to Ph. Eur.

To increase the porosity of the basic shape’s outline, we designed the segment model of the tablet with one outline containing two hollow segments on the longer sides, and pores with a size of a minimum of 0.8 mm × 1.64 mm on the arc parts of the tablet. The pores were created by adding small channels, which can be seen in Figure 2 (segments; top view). The calculated surface area/mass ratio of DOM_segments and PAR_segments formulations increased (Table 4), but the disintegration time presented in Figure 3 was surprisingly longer than in the case of the basic shape. The channels were designed to be hollow, but due to the printer’s nozzle diameter of 0.2 mm and thicker printed path caused by Barus effect, the opposite effect occurred: dense and solid channels were created which disintegrated the longest.

Another approach was to increase the surface area of the outline by designing a crown shape containing two different sizes of the “crown”, a smaller (inner) and a larger (outer) one, which altered in each layer. This led to (a) the creation of new channels through which the disintegration medium could flow, and (b) the reduction of layer contact from full to point contacts at the corners of the structure. This design resulted in increasing the surface/mass ratio and decreasing the tablet disintegration time, both in PAR_crown and DOM_crown formulations (Figure 3, disintegration times equal to 3 min 10 s ± 19 s and 9 min 5 s ± 23 s for PAR_crown and DOM_crown tablets, respectively).

The next option was to omit the outline completely and prepare tablets only with the 10% and 15% infill. This led to the preparation of porous structures with layers laying on each other in a rectangular manner. The only parameter that stayed constant was the tablet mass; therefore, the number of layers in each shape had to be increased to reach the desired mass and dosage. The “15% infill” and “10% infill” shapes were characterized by a further increase in the surface area: in the case of these formulations, the surface/mass ratio was equal to or greater than 1, while for “basic” tablets it was equal to 0.81 (Table 4). The fastest disintegration for formulations made of the DOM filament was observed in the case of DOM_15% tablets (7 min 55 s ± 8 s, Figure 3B). However, the disintegration time of PAR_15% tablets, equal to 3 min 18 s ± 24 s, was comparable to the PAR_crown (Figure 3A). In the case of 10% infill shape, the height of the tablet was too high (6.3 mm), and overall, the size of the tablet was too big, which resulted in prolongation of water penetration and thus, the disintegration time.

#### 2.3.3. The Effect of Composition—The Addition of Mannitol

All formulations containing the API, crospovidone, and PVA failed to disintegrate within 3 min. We consequently decided to add 10% of mannitol as a plasticizer and a pore-former to the filament composition. By intercalating between the branches of the polymer, the plasticizer reduces the interaction between molecules, thereby it increases mobility and improves the flowability of the material, which is beneficial for both hot-melt extrusion and the printing process [40]. In addition, the water solubility of mannitol increases the proportion of hydrophilic substances in the filament composition, which was believed to promote the tablets’ disintegration.

Furthermore, in the case of tablets with domperidone, the API content in filament was increased from 10 to 20%, which allowed us to decrease the desired tablet mass from 100 mg to 50 mg while maintaining the same 10 mg dose. The crown, infill 10%, and infill 15% shapes exhibited the highest surface/mass ratios together with the shortest disintegration time, which was proven in the previous tests of plasticizer-free tablets, hence, they were selected for the preparation of formulations with mannitol.

From the graphs presented in Figure 3, we can note that the addition of mannitol led to a decrease in disintegration time for PAR + M tablets with infill 10% and 15% (disintegration times 2 min 34 s ± 5 s and 2 min 22 s ± 2 s, respectively). In the case of tablets made of DOM + M filament, there was an acceleration of disintegration for all tested tablet shapes, with the crown shape disintegrating within 2 min 25 s ± 1 s, thus fulfilling the pharmacopeial limit of 3 min.

### 2.4. Comparison of the Tablets’ Microstructure with 3D Models

#### 2.4.1. Scanning Electron Microscopy

In order to compare the degree of mapping of the tablet structure, we compared the tablets designs with pictures obtained by scanning electron microscopy. The analysis was performed for the formulations characterized by the shortest disintegration time and for the corresponding tablets without mannitol, i.e., PAR_15%, PAR + M_15%, DOM_crown, and DOM + M_crown.

**Figure 4:**
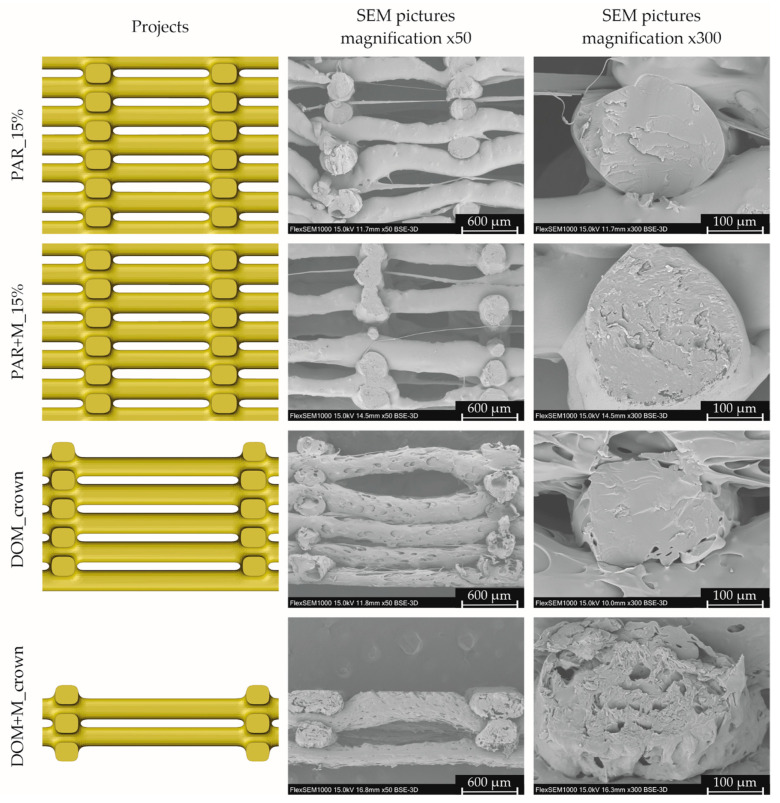
Tablets cross-sections based on designs received by reversed voxelization and SEM pictures (magnification 50× and 300×).

The structures of both tablets made of filaments containing paracetamol, visualized by a scanning electron microscope, are similar (Figure 4). Extruded paths have irregular diameters, in some places consisting only of thin fibers. Although the extruded paths were not regular, they did not merge between their intersections, and the porous structure of the tablet has been reproduced. Moreover, tablets made of PAR, as well as PAR + M filaments, were characterized by a smooth surface and homogenous paths’ infill without voids.

The structure of tablets made of filaments containing domperidone was more regular. All paths visible in the tablets’ cross-sections had a similar height of approx. 260 µm, while the path height set in the project was equal to 150 µm. The difference between the real and theoretical layer height could be caused by the paths’ deformation (paths were not supported between intersections) and the Barus effect, related to the material swelling after being extruded through the printer nozzle.

In the case of tablets made of the DOM filament, most of the paths visible in cross-section were bent, which made the structure less porous compared to the project. The surface of both tablets containing domperidone was rough and porous. For both formulations, pores were located in the entire volume of the extruded paths, which was visible in cross-sections. In the case of tablets made of DOM + M filament, pores were smaller in comparison to the formulation without mannitol. This porous structure can facilitate water penetration and thus accelerate the tablets’ disintegration. Additional SEM pictures of 3D printed tablets are attached to the Supplementary Materials.

#### 2.4.2. 3D Microscopy

**Figure 5:**
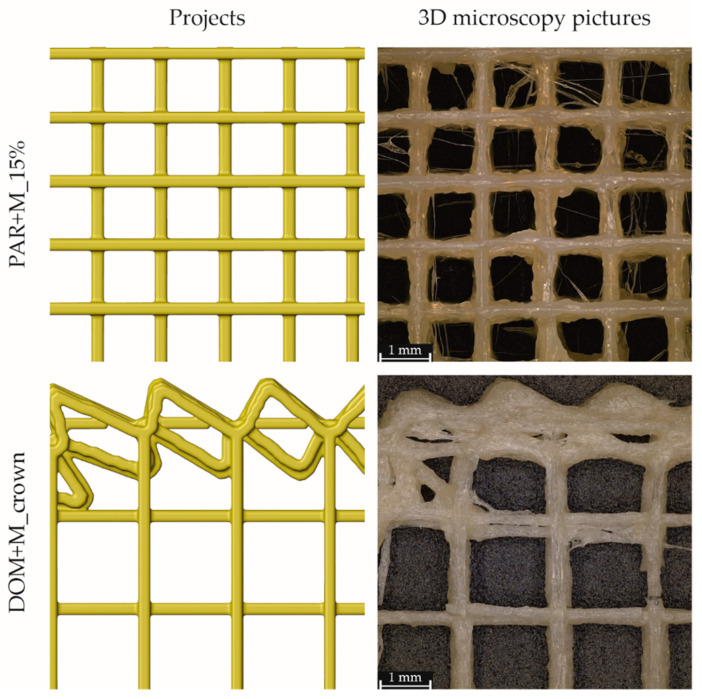
Top views on PAR + M_15% and DOM_crown tablets: comparison of designs received by reversed voxelization (**left**) and obtained by 3D microscopy (**right**).

For the formulations characterized by the fastest disintegration (PAR + M_15% and DOM + M_crown), we also compared the designs with the top views obtained from 3D microscopy, presented in Figure 5. Similar to the height of the printed paths, the width was also greater than the theoretical one. For the PAR + M_15% tablets, the measured path width was approximately 20% greater, while for tablets made of DOM + M filament—more than two times greater than the set one.

The structure of tablets containing paracetamol and mannitol was irregular: the material was unevenly extruded from the printer nozzle and slightly deformed, and fine fibers are visible between the main structure of the mesh. Despite slight deformation, the structure of the mesh was reproduced: the lines of the mesh were separated and formed open pores that allowed the medium to pass through the tablets. In the case of DOM + M_crown tablets, the structure of the porous infill was reproduced. Although the paths forming the crown were partially deformed and the surface area of connection was greater in comparison to the project, small pores were still visible.

### 2.5. Atomic-Scale Structure Analysis

**Figure 6:**
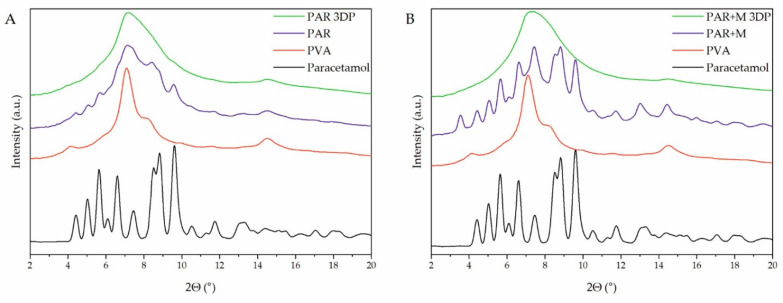
XRPD diffractograms of raw paracetamol, PAR (**A**) and PAR + M (**B**) filaments, and 3D printed tablets made of PAR (**A**) and PAR + M (**B**) filaments.

X-ray powder diffraction (XRPD) was utilized to analyze the atomic-scale structure of raw materials, extrudates, and 3D printed tablets. The diffractogram of raw paracetamol (Figure 6A,B, black lines) shows characteristic sharp Bragg peaks indicating its crystalline structure. The analysis of the extrudates revealed that paracetamol was partially in a crystalline form after the hot-melt extrusion process, as the sharp peaks at the same positions as for raw API can be observed in the filaments diffractograms (Figure 6A,B, violet lines). A higher degree of crystallinity of paracetamol can be observed for extrudates containing the addition of mannitol due to its plasticizing effect. This is caused by the low temperature of the hot-melt extrusion process which was conducted below the melting point of the paracetamol (Tm = 168 °C, based on the DSC measurements). Moreover, in the diffractogram of extrudate with mannitol in Figure 6B, a weak peak around 3.5° appears, which results from the recrystallization of mannitol. After printing processes carried out at 183 °C and 165 °C for systems without and with the addition of mannitol, respectively, the samples were amorphized. There are no signs of recrystallization of API or any other component of the formulation in the diffractograms of 3D printed tablets. The maxima observed in the diffractograms for the 3D printed tablets (Figure 6A,B, green lines) are characteristic of PVA, which is the main component and has a semicrystalline nature.

**Figure 7:**
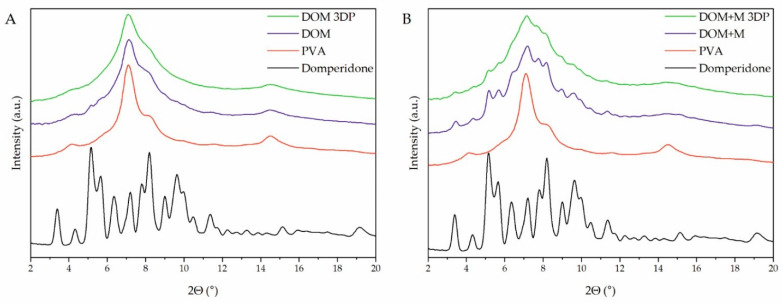
XRPD diffractograms of raw domperidone, DOM (**A**) and DOM + M (**B**) filaments, and 3D printed tablets made of DOM (**A**) and DOM + M (**B**) filaments.

In the case of domperidone-containing formulations, only the diffractogram obtained for 3D printed tablets without the addition of mannitol (Figure 7A, green line) shows no signs of recrystallization. All tested extrudates, as well as DOM + M 3D printed tablets, contained a fraction of crystalline domperidone. The filaments were extruded below the melting temperature of domperidone (Tm = 247 °C, based on the DSC measurements) while the 3D-printing process was conducted at 250 °C and 220 °C for DOM and DOM + M formulations, respectively. The high temperature of the DOM printing process efficiently induced its amorphization. The results of the DSC analyses confirmed the results obtained with XRPD and are presented in the Supplementary Materials.

### 2.6. Raman Mapping

**Figure 8:**
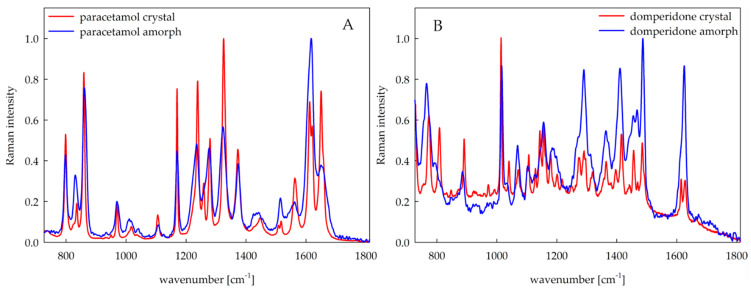
Reference Raman spectra of paracetamol in crystalline and amorphous form (**A**) and domperidone in crystalline and amorphous form (**B**).

Raman mapping was used to study the core of the 3D printed tablets. Thanks to the precise focusing of the laser, this method enables a detailed spatially resolved analysis of the tablet. Since a solid solution was used to produce the tablets, the individual components of the tablets should not be distinguished, as the Raman spectra should be identical at each tablet location. Thus, using Raman mapping, it was possible to investigate the homogeneity of the tablets and the presence of a crystalline or amorphous form of the APIs (Figure 8A,B for paracetamol- and domperidone-loaded tablets, respectively), since the excipients used for tablet production have a weak Raman signal. This makes the APIs signal relatively more intense, and therefore, it was possible to investigate the presence of APIs.

**Figure 9:**
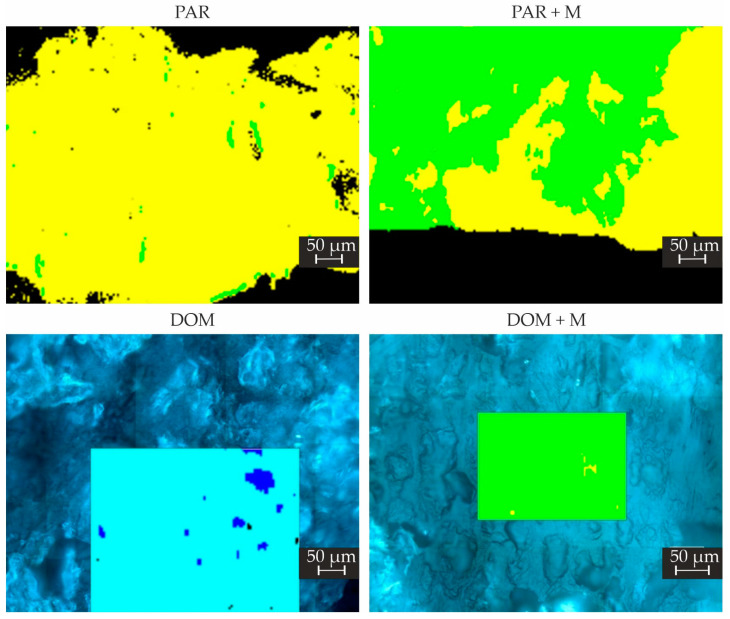
Raman maps of tablets made of filaments with different compositions with 50× magnification and 4 μm step used. Yellow is for APIs in amorphous form, green for crystalline, blue for crospovidone, cyan for PVA, and black for wax or unidentified spectra.

A large predominance of amorphous API was observed in tablets made of PAR filament with a small amount of crystalline form on a few small locations of the tablet. In contrast, both forms of paracetamol were confirmed in the case of tablets composed of PAR + M filament with similar abundance (Figure 9).

In the case of tablets made of DOM + M filament, the crystalline form of API was found to be highly predominant over the amorphous form (Figure 9). Since the API content in printlets made of DOM formulation was lower than in paracetamol tablets, the Raman signal of API in the amorphous form was less intense but visible throughout the tablet cross-section. However, some inhomogeneity of excipients content was observed in the tablets made of DOM filament because of the presence of locations with a predominance of crospovidone or PVA over the other tablet components. The additional Raman maps for tested formulations are included in the Supplementary Materials.

The results slightly differ from the XRPD and DSC results. The reason is that the Raman maps were created by using a step size of 4 μm. Since there is no preponderance of crystals in the tablets, even small amounts can be detected by Raman mapping on the surface, but not by XRPD and DSC, where micrograms of the sample were used.

### 2.7. Drug Dissolution Studies

Disintegrants are widely used in the solid oral dosage form technology to impair tablet structure. In our previous works, crospovidone, which disintegrates tablets by swelling without gel formation, proves to be an effective excipient to improve the dissolution rate of API from 3D printed tablets [18]. Additionally, mannitol, which was used as a plasticizer, can also act as a channeling agent and positively influence the drug dissolution form of polymeric 3DP tablets [41,42].

**Figure 10:**
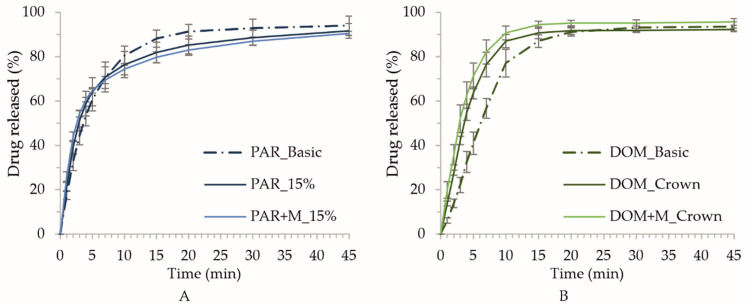
Dissolution profiles of paracetamol (**A**) and domperidone (**B**) from 3DP tablets.

The dissolution profiles show that all tested printlets behave as immediate release dosage forms. It is worth mentioning that over 80% of paracetamol and domperidone was released from the printlets within the first 15 min of the study (Figure 10A,B for paracetamol- and domperidone-loaded tablets, respectively). Nevertheless, the higher amount of API was released from tablets containing domperidone in comparison to the paracetamol-loaded formulation. After 10 min over 85% of domperidone was released from crown like tablets whereas paracetamol printlets released between 75% and 80% of API. These differences are related to the porous structure of domperidone printlets, which is clearly visible on SEM images (Figure 4).

## 3. Materials and Methods

### 3.1. Materials

Paracetamol (*N*-(4-hydroxyphenyl)acetamide) granules (96% API content) manufactured by fluid bed granulation with polyvinylpyrrolidone, supplied by Anqiu Lu’an Pharmaceutical Co., Ltd. (Anqiu, China) and Domperidone (6-chloro-3-[1-[3-(2-oxo-3*H*-benzimidazol-1-yl)propyl]piperidin-4-yl]-1*H*-benzimidazol-2-one) (Sri Krishna Pharmaceuticals, Ltd., Hyderabad, Telangana, India) served as model active ingredients. Poly(vinyl alcohol) (PVA, Parteck^®^ MXP, Merck^®^ KGaA, Darmstadt, Germany) was used as the matrix-forming polymer to prepare filaments and 3D printed tablets. Crospovidone (Kollidon^®^ CL, BASF^®^, Ludwigshafen, Germany) was utilized as a disintegrant, while Mannitol (Avantor^®^ Performance Materials Poland S.A., Gliwice, Poland) was added to plasticize the formulations. Hydrochloric acid solution (Merck^®^ KGaA, Darmstadt, Germany) was used to prepare the medium for drug-loading and dissolution tests of domperidone-loaded tablets. The water used in all tests was produced by Elix 15UV Essential reversed osmosis system (Merck^®^ KGaA, Darmstadt, Germany).

### 3.2. Preparation of Drug-Loaded Filaments

The filament extrusion processes were performed using a 40D length, 12-mm co-rotating twin-screw extruder (RES-2P/12A Explorer, Zamak Mercator^®^, Skawina, Poland) equipped with gravimetric feeder (MCPOWDER^®^ Movacolor^®^, Sneek, The Netherlands), an air-cooled conveying belt (Zamak Mercator^®^, Skawina, Poland) and two-dimensional laser diameter gauge (LDM25XY, Mercury-Tech Co., Ltd., Zhengzhou, China). The mixtures of API, filament-forming polymer and additives were prepared by geometric dilution prior to the extrusion process. The powder blends were homogenized and plastified in the barrel of the extruder and then extruded through a 1.75 mm die at a temperature adjusted to the properties of the mixture. The utilized screw design has been described in our previous work [18].

### 3.3. Evaluation of Filament Diameter and Mechanical Properties

The filaments’ diameter uniformity was evaluated using a Mitutoyo^®^ micrometer screw (Kawasaki, Tokyo, Japan). Mechanical properties were evaluated in a stretching test performed with an EZ-SX tensile tester (Shimadzu^®^, Kioto, Japan) equipped with a 500 N load cell. The measurements were performed for six randomly selected pieces of each filament. Samples were placed in the tensile tester’s jaws and stretched up with 1000 mm/min speed to breakage. The gauge length was equal to 50 mm, and the precisely measured diameter of the tested specimens was included to evaluate the hardness and elasticity of the filaments.

### 3.4. Determination of Drug Content in the Filaments

Three randomly selected and accurately weighed pieces of each filament were placed in conical flasks filled with 50 mL of water or 0.1 M HCL for paracetamol- and domperidone-loaded filaments, respectively. Next, samples were shaken for 24 h in a Memmert^®^ water bath (WNB 22, Schwabach, Germany) and filtered through the CHROMAFIL^®^ Xtra CA-45/25 syringe filters. The concentration of APIs was determined spectrophotometrically at 243 nm for paracetamol and 284 nm for domperidone using a Jasco V-530 spectrophotometer (Tokyo, Japan). The specificity of the analytical method was verified; there was no sign of interference between the drugs and excipients at the analytical wavelengths.

### 3.5. Tablets Design and Surface Analysis

We created and analyzed tablets with five shapes characterized by very porous architectures. The basic shapes of the tablets were designed using Blender 2.90 software (Blender Foundation, Amsterdam, The Netherlands). All tablets were created based on the project of oblong tablets 10 mm wide and 20 mm long. Then, projects were exported to Voxelizer slicing software (version 1.4.18, Zmorph S.A., Wroclaw, Poland) to create precise models of tablets and calculate the amount of filament needed to print tablets. The following settings were applied for the slicing process: 0.15 mm of layer height, 1.75 mm of the theoretical filament diameter, and outline and infill settings appropriate for the specific shapes. The path width corresponding to nozzle diameter was set to 0.2 mm. The height of the tablets was determined based on Voxelizer calculations of the required amount of filament and ranged from 0.75 mm to 6.30 mm to obtain the targeted dosage of APIs, depending on the tablets’ shapes and drug-loading in filaments.

Next, the generated tablets projects were used to calculate the surface areas. The precise models were imported into Voxelizer 2 slicing software (version 2.0.0, Zmorph S.A., Wroclaw, Poland) to perform reversed voxelization process to determine the 3D structures based on gcodes. The process was performed with a 0.02 voxel size setting, which was selected experimentally to accurately reproduce the actual structure of the tablets. The surface areas of the tablets were calculated in the Blender software.

### 3.6. Preparation of 3D Printed Tablets

The tablets were printed by FDM ZMorph^®^ 2.0 S personal fabricator (Wroclaw, Poland) equipped with a 1.75 mm commercially available printhead with 0.2 mm nozzle. All tablets were printed with the same printing speed equal to 10 mm/s. The remaining printing parameters were the same as used for the preparation of the gcodes for surface areas calculations. The melted filaments were deposited by printing toolhead onto the printing table covered by the COROPad™ adhesive pad (HMF Chemicals, Grodzisk Mazowiecki, Poland) warmed up to 65 °C. The printing temperature ranged from 165 to 250 °C, depending on the composition of the filament. The differences between the actual and theoretical diameters of the filaments were compensated by changing the filament diameter settings in the slicing software. All tablets were weighed immediately after printing. The basic-shaped tablets were stored for 24 h at room temperature, in a desiccator, or a climate chamber (25 °C, RH 60%), to evaluate the effect of storage conditions on disintegration time. Tablets for all other tests were stored in the desiccator.

### 3.7. Disintegration Test

Three printed tablets from each formulation were tested for disintegration time employing SAPO ED-2 apparatus (Electrolab, Mumbai, India) without the application of discs. 700 mL of purified water heated to 37 ± 0.5 °C was used as a disintegration medium. The mean and standard deviation were calculated from the values obtained.

### 3.8. Differential Scanning Calorimetry (DSC)

Thermodynamic properties of raw materials, filaments, and 3D printed tablets were examined using a DSC 1 STARe System (Mettler-Toledo^®^, Greifensee, Switzerland). The apparatus was equipped with an HSS8 ceramic sensor (heat flux sensor with 120 thermocouples) and liquid nitrogen cooling accessory. The instrument was calibrated for temperature and enthalpy using indium and zinc standards. The samples were measured in 40 µL pinned aluminum pans in a nitrogen atmosphere. All measurements were carried out with gas flow equal to 60 mL/min and heating rate 10 K/min. Melting points of the samples were determined as an onset of the peaks, and glass transition temperatures were determined as the midpoint of the heat capacity increment.

### 3.9. X-ray Powder Diffraction (XRPD)

X-ray diffraction experiments were performed using a Rigaku–Denki D/MAX RAPID II-R diffractometer (Rigaku Corporation, Tokyo, Japan) with a rotating Ag anode, an incident beam (002) graphite monochromator, and a two-dimensional image plate detector in the Debye–Scherrer geometry. The incident beam’s wavelength was λ = 0.5608 Å. The pixel size was 100 μm × 100 μm. Samples were measured in borosilicate glass capillaries of 1.5 mm in diameter. The beam width of the sample was 0.3 mm. The angular range covered by the experiment was 2Θ: 2–164° with a step size equal to 0.045°. The two-dimensional diffraction patterns were corrected for background and converted into one-dimensional intensity data versus the scattering angle 2Θ.

### 3.10. Raman Mapping

To prepare the 3D tablets for Raman mapping, one tablet of each kind was sealed in the paraffin block and cut on a Leica RM2255 rotary microtome (Leica Biosystems Nussloch GmbH, Nussloch, Germany) several times with a step of 2 μm to reach approximately the middle of the tablet and to obtain a section as smooth as possible to avoid spectral artifacts. Subsequently, the Raman maps were obtained using the dispersive inVia Reflex Raman microscope (Renishaw, Wotton-under-Edge, UK) coupled with an integrated Leica microscope and equipped with a high sensitivity ultra-low noise CCD detector Renishaw RenCam (Renishaw, Wotton-under-Edge, UK), an XYZ motorized stage and an excitation diode laser at a wavelength of 785 nm. The measurements were performed using a lens with a magnification of 50 (0.75 N.A.), a monochromator utilized dual gratings of 1200 grooves/mm, a resolution of 0.5−1 cm^−1^ and an exposure time of 0.1 and 1 s for tablets with paracetamol and domperidone, respectively. For domperidone tablets, a longer exposure time was set to reduce the luminescence of the tablets to obtain better spectra. The incident laser power on the sample was approximately 30 mW. To avoid information bias, each tablet was measured at several different locations. A sample area was approximately 860 × 550 μm and 400 × 320 μm for paracetamol and domperidone tablets, respectively. For the mapping of domperidone tablets, a smaller sampling area was chosen due to the longer exposure time for a single spectrum and thus longer analysis duration. A step size of 4 μm was used, and the spectral range was set to 727–1812 cm^−1^. 

The data pre-processing, including the normalization of the raw spectra and the cosmic ray removal of spectral artefacts, were performed in WiRE 5.4 program (Renishaw, Wotton-under-Edge, UK). No additional smoothing or baseline correction was performed.

To evaluate the Raman maps, a direct classical least squares (DCLS) algorithm was employed. For this method, only the reference spectra of the pure APIs and excipients were necessary. The DCLS method sought a linear combination of the reference spectra to find the best match with each point (each spectrum) in the map. In this way it was possible to construct Raman maps of the tablets.

### 3.11. Scanning Electron Microscopy (SEM)

The microstructure of the 3D printing filaments and the 3D printed tablets was examined using a scanning electron microscope FlexSEM 1000 (Hitachi, Tokyo, Japan) working in secondary electrons mode and backscattered electrons mode at an accelerating voltage of 15 kV. The top surfaces and the cross-section surfaces of the filaments before the print were imaged. Furthermore, the arrangement and morphology of filaments in the 3D printed tablets were observed: For the formulations containing domperidone and domperidone with mannitol, the tablets of geometry “Crown” were studied, while for the formulations containing paracetamol and paracetamol with mannitol, the tablets of the “Infill 15%” geometry were examined. Prior to the measurements, all samples were attached to a carbon adhesive tape and coated with an 8 nm thick layer of gold by EM ACE200 sputter coater (Leica Microsystems, Wetzlar, Germany).

### 3.12. 3D Microscopy

The 3D photos of tablets were taken using an Olympus DSX1000 optical microscope (Olympus, Tokyo, Japan) equipped with a DSX10-SZH zoom head, DSX10-XLOB Plan FL 3× (0.09 N.A.) long working distance objective lens, DSX10-RMTS motorized XY stage with rotation function, and XDSX10-TF tilting frame. Printed tablets without any treatment were placed on a XY stage and 3D photos were taken with a darkfield observation mode and magnification on a monitor of 42×, 150× or 300× using DSX software (Olympus, Tokyo, Japan).

### 3.13. Drug Dissolution Studies

Dissolution studies were performed in accordance with Ph. Eur. 10 in the type II (paddle) apparatus (Hanson Research SR8 Plus with autosampler Dissoette II, Chatsworth, CA, USA) at 50 rpm. The 3DP tablets were inserted into the Ph. Eur. sinkers and tested in 1000 mL of 0.1 M HCl in the case of domperidone tablets or in deionized water in the case of paracetamol tablets at a temperature of 37 °C. Samples were withdrawn at 1, 2, 3, 4, 5, 7, 10, 15, 20, and 30 min filtered through 10 μm filters and analyzed spectrophotometrically with UV-VIS Jasco V-530 spectrophotometer (Tokyo, Japan) equipped with a flow-through cuvettes: 10 mm for domperidone analysis or 1 mm for paracetamol analysis. The samples were analyzed at λ = 284 and λ = 243 nm, respectively. The tests were carried out in six repetitions (*n* = 6) and the results represent averages with their standard deviations (SD).

## 4. Conclusions

This study proved the feasibility of preparing orodispersible tablets using fused deposition modeling. We investigated the effect of different storage conditions, spatial structures, and the presence of mannitol on tablet parameters.

Filaments containing paracetamol or domperidone, polyvinyl alcohol as a matrix-forming polymer, crospovidone as a superdisintegrant supporting the structure disruption, and eventually the mannitol as a plasticizer were prepared by hot-melt extrusion. All filaments contained the desired percentage of the APIs, exhibited proper tensile strength, and were printable after setting the correct printing temperatures for each formulation individually. XRPD and DSC analysis revealed that all extrudates, regardless of the mannitol content, comprised the API in crystalline form. However, the printing process induced amorphization in the case of all tablets containing paracetamol and those containing domperidone without mannitol. These results were supported and visualized by Raman mapping with slight discrepancies.

Five different spatial shapes, i.e., basic, segments, crown, infill 10%, and infill 15%, were designed and successfully printed with the content of paracetamol (100 mg) and domperidone (10 mg) therapeutic doses. The latter three shapes exhibited the highest surface/mass ratio and therefore were selected for the preparation of tablets containing formulations with mannitol. The disintegration time test subsequently showed that the crown and infill 15% tablets for domperidone and paracetamol, respectively, reached the shortest disintegration time fulfilling the Ph. Eur. limit. Hence, the addition of mannitol decreased the printing temperature and promoted tablet disintegration due to its water-soluble nature. The SEM and 3D microscopy images showed that the porous structure of these tablets was reproduced. Furthermore, the domperidone formulations contained voids inside the printed paths, which could have been responsible for the accelerated disintegration. The formulations with the shortest tablet disintegration time showed the immediate release profiles, with 85% of domperidone and 75% of paracetamol released within 10 min.

## Figures and Tables

**Table 1:** The composition and extrusion temperature of filaments.

Formulation	API	API Content	PVA	PVP CL	Mannitol	Die Temp.
PAR	Paracetamol	40%	55%	5%	-	155 °C
PAR + M			45%		10%	145 °C
DOM	Domperidone	10%	85%		-	200 °C
DOM + M		20%	65%		10%	190 °C

**Table 2:** Hot-melt extruded filaments characteristics (SD is the standard deviation).

Formulation	API Content ± SD (%)	Diameter ± SD (mm)	Tensile Strength ± SD (MPa)	Young’s Modulus ± SD (MPa)
PAR	41.36 ± 0.18	1.71 ± 0.06	15.34 ± 2.51	566.04 ± 80.80
PAR + M	41.78 ± 0.37	1.77 ± 0.05	10.66 ± 1.78	873.18 ± 95.06
DOM	10.35 ± 0.22	2.08 ± 0.09	29.67 ± 3.25	1597.67 ± 109.75
DOM + M	20.57 ± 0.35	2.16 ± 0.06	12.98 ± 4.89	834.47 ± 12.20

**Table 3:** Comparison of the attributes of tablets with basic shape immediately after printing and after 24 h of storage in various conditions.

Filament	Weight after Printing (mg) ± RSD (%)	Weight after Storing (mg) ± RSD (%)	Weight Change (%)	Disintegration Time (min:s) ± SD
Climate chamber (25 °C, 60% RH)				
PAR	252.36 ± 2.91	266.70 ± 2.79	+5.68	8:02 ± 0:21
DOM	100.67 ± 1.45	108.31 ± 1.36	+7.59	8:46 ± 1:49
Room conditions (22.7 °C, 60.6% RH)				
PAR	258.72 ± 0.60	268.80 ± 0.34	+3.90	5:46 ± 1:11
DOM	98.33 ± 2.69	105.17 ± 3.13	+6.96	11:43 ± 2:04
Desiccator (22.7 °C, 35% RH)				
PAR	246.24 ± 2.97	245.52 ± 2.91	−0.30	4:28 ± 0:07
DOM	95.57 ± 2.06	95.42 ± 1.99	−0.15	9:54 ± 0:17

**Table 4:** Attributes of tablets based on projects obtained by reversed voxelization.

Sample Name	Filament & Dosage (mg)	Tablets Shape	Tablets Height (mm)	Surface Area (mm^2^)	Surface/Mass Ratio (mm^2^/mg)	Printed Tablets Mass (mg) ± RSD (%)
PAR_basic	PAR 100.0	Basic	4.20	201.78	0.81	246.24 ± 2.97
PAR_segments		Segments	4.95	231.81	0.93	256.72 ± 1.87
PAR_crown		Crown	3.60	239.17	0.96	258.94 ± 0.36
PAR_10%		Infill 10%	6.30	250.93	1.00	241.37 ± 2.79
PAR_15%		Infill 15%	4.80	280.46	1.12	252.29 ± 2.87
PAR + M_crown	PAR + M 100.0	Crown	3.60	239.17	0.96	253.70 ± 3.76
PAR + M_10%		Infill 10%	6.30	250.93	1.00	251.78 ± 3.83
PAR + M_15%		Infill 15%	4.80	280.46	1.12	247.00 ± 1.84
DOM_basic	DOM 10.0	Basic	1.65	81.40	0.81	95.57 ± 2.06
DOM_segments		Segments	1.80	88.14	0.88	100.60 ± 4.88
DOM_crown		Crown	1.50	105.61	1.06	100.67 ± 4.90
DOM_10%		Infill 10%	2.55	101.58	1.02	99.73 ± 5.35
DOM_15%		Infill 15%	1.95	114.92	1.15	102.04 ± 4.40
DOM + M_crown	DOM + M 10.0	Crown	0.75	53.78	1.08	52.19 ± 7.45
DOM + M_10%		Infill 10%	1.35	54.20	1.08	48.64 ± 6.16
DOM + M_15%		Infill 15%	1.05	62.55	1.25	55.55 ± 2.23

## Data Availability

Data is contained within the article and Supplementary Material.

## Supplementary Materials

The following are available online at https://www.mdpi.com/article/10.3390/ph15010069/s1, Figure S1: Cross-sections of filaments analyzed by SEM: (a)—PAR, (b)—PAR + M, (c)—DOM, (d)—DOM + M (300× magnification), Figure S2: Surfaces of 3D printed tablets analyzed by SEM (magnification 50×, 100× or 300×), Figure S3: DSC thermograms obtained for APIs (A) and excipients (B), Figure S4: DSC thermograms obtained for filaments (A) and 3D printed tablets (B) with domperidone, Figure S5: DSC thermograms obtained for filaments (A) and 3D printed tablets (B) with paracetamol, Figure S6: Raman maps of tablets made of filaments with different compositions with 50× magnification and 4 μm step used. Yellow is for APIs in amorphous form, green for crystalline, blue for crospovidone, cyan for PVA, and black for wax or unidentified spectra.
