# Supplementary material for: Fused Deposition Modeling as a Possible Approach for the Preparation of Orodispersible Tablets
Source: Pharmaceuticals (Basel). 2022 Jan 5;15(1):69. doi: 10.3390/ph15010069 (PMC8781976; doi:10.3390/ph15010069)
Supplement: Supplementary file 1 [file pharmaceuticals-15-00069-s001.zip › pharmaceuticals-1507950-supplementary.pdf]

pharmaceuticals

Supplementary Materials

Fused Deposition Modeling as a Possible Approach for the Preparation of Orodispersible Tablets

Thao Tranová [1], Jolanta Pyteraf [2,*], Mateusz Kurek [2], Witold Jamróz [2], Witold Brniak [2], Dita Spálovská [3,4], Jan Loskot [5], Karolina Jurkiewicz [6], Joanna Grelska [6], Daniel Kramarczyk [6], Jitka Mužíková [1], Marian Paluch [6] and Renata Jachowicz [2]

1. Department of Pharmaceutical Technology, Charles University, Faculty of Pharmacy in Hradec Králové, Akademika Heyrovského 1203, 500 05 Hradec Králové, Czech Republic; tranthip@faf.cuni.cz (T.T.); muzikova@faf.cuni.cz (J.M.)
2. Department of Pharmaceutical Technology and Biopharmaceutics, Jagiellonian University Medical College, Medyczna 9, 30-688 Krakow, Poland; mateusz.kurek@uj.edu.pl (M.K.); witold.jamroz@uj.edu.pl (W.J.); w.brniak@uj.edu.pl (W.B.); renata.jachowicz@uj.edu.pl (R.J.)
3. Department of Analytical Chemistry, University of Chemistry and Technology Prague, Technická 5, 166 28 Prague 6, Czech Republic; dita.spalovska@vscht.cz (D.S.)
4. Zentiva, k.s, U Kabelovny 130, 102 37 Prague 10, Czech Republic
5. Department of Physics, University of Hradec Králové, Faculty of Science, Rokitanského 62, 500 03 Hradec Králové, Czech Republic; jan.loskot@uhk.cz (J.L.)
6. A. Chełkowski Institute of Physics, University of Silesia in Katowice, ul. 75 Pułku Piechoty 1, 41-500 Chorzów, Poland; karolina.jurkiewicz@us.edu.pl (K.J.); joanna.grelska@us.edu.pl (J.G.); daniel.kramarczyk@smcebi.edu.pl (D.K.); marian.paluch@us.edu.pl (M.P.)

* Correspondence: jolanta.pyteraf@uj.edu.pl; Tel.: +48-12-62-05-600

Citation: Tranová, T.; Pyteraf, J.; Kurek, M.; Jamróz, W.; Brniak, W.; Spálovská, D.; Loskot, J.; Jurkiewicz, K.; Grelska, J.; Kramarczyk, D.; et al. Fused Deposition Modeling as a Possible Approach for the Preparation of Orodispersible Tablets. *Pharmaceuticals* **2022**, *15*, 69. https://doi.org/10.3390/ph15010069

Academic Editors: Touraj Ehtezazi and Rachel Auzély

Received: 29 November 2021
Accepted: 2 January 2022
Published: 5 January 2022

Publisher's Note: MDPI stays neutral with regard to jurisdictional claims in published maps and institutional affiliations.

Copyright: © 2022 by the authors. Submitted for possible open access publication under the terms and conditions of the Creative Commons Attribution (CC BY) license (https://creativecommons.org/licenses/by/4.0/).

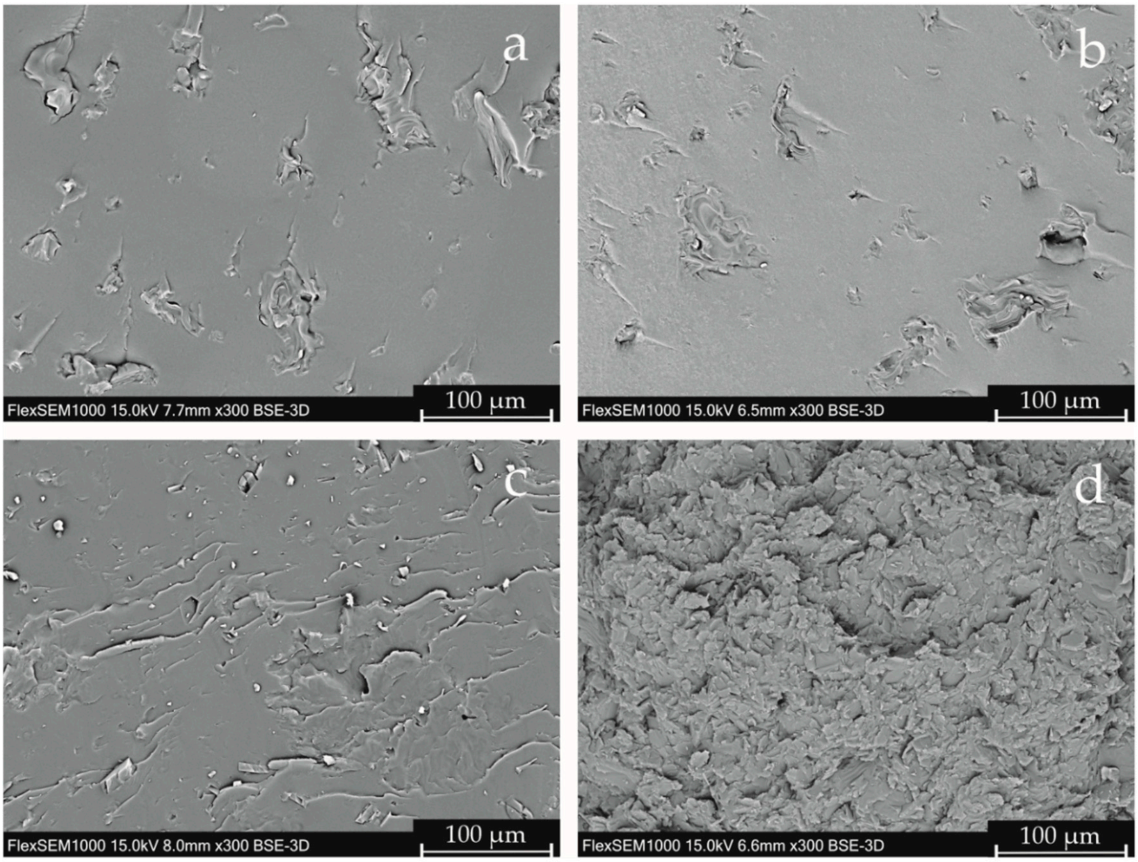

Figure S1 Cross-sections of filaments analyzed by SEM: a – PAR, b – PAR+M, c – DOM, d – DOM+M (300x magnification).

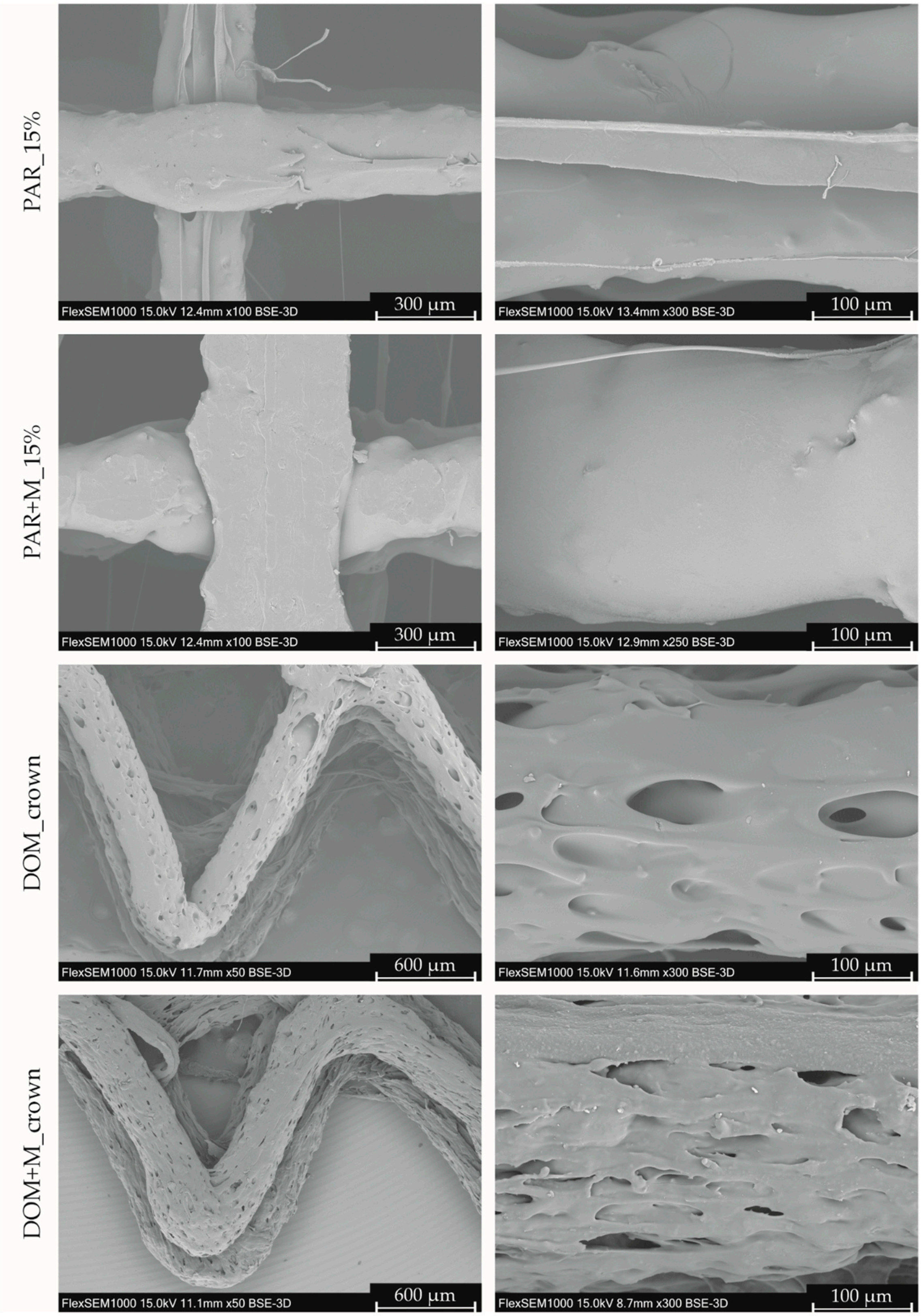

Figure S2 Surfaces of 3D printed tablets analyzed by SEM (magnification 50x, 100x or 300x).

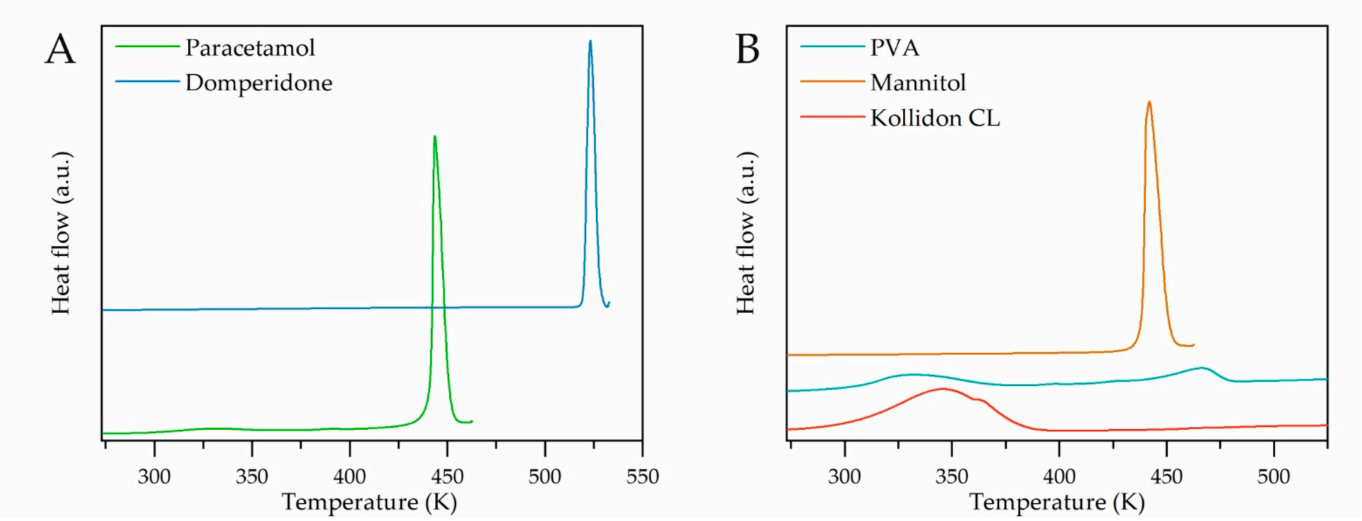

Figure S3 DSC thermograms obtained for APIs (A) and excipients (B).

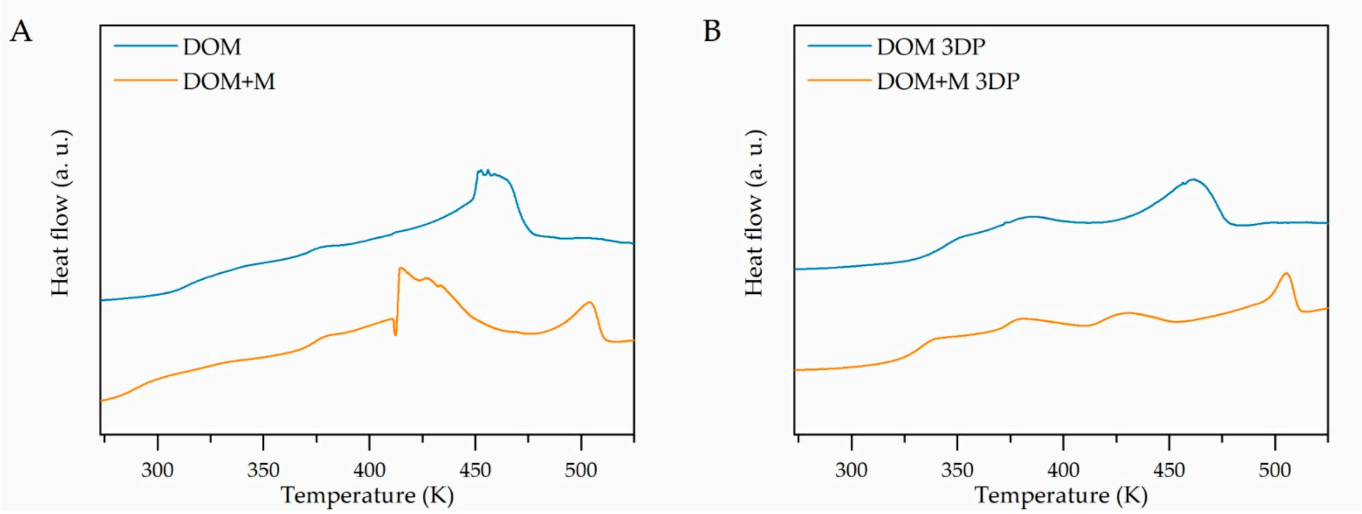

Figure S4 DSC thermograms obtained for filaments (A) and 3D printed tablets (B) with domperidone.

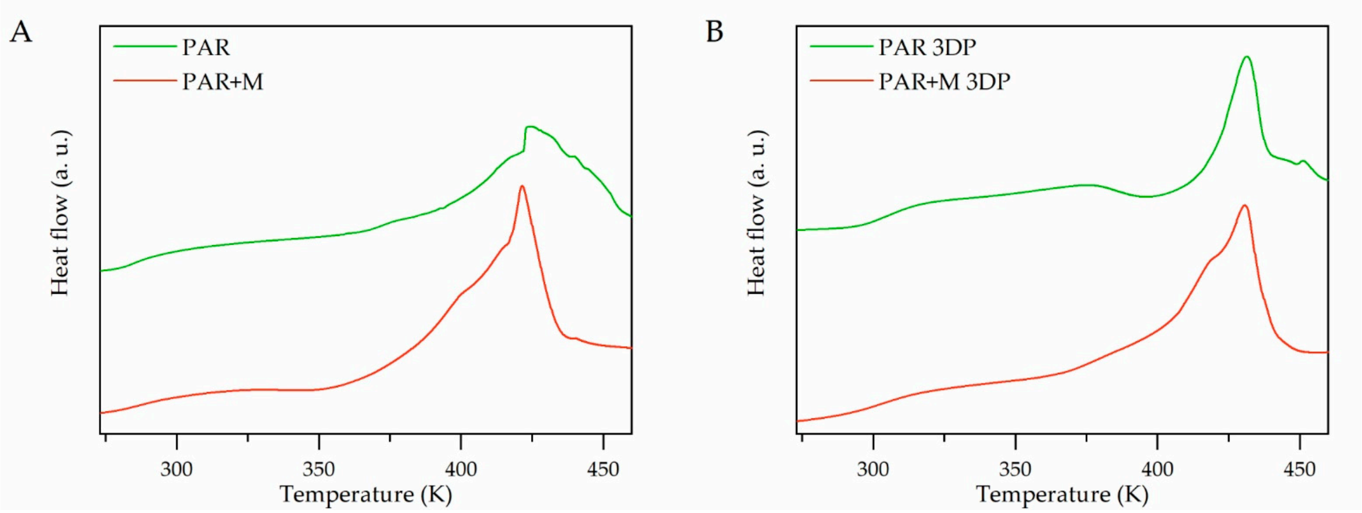

Figure S5 DSC thermograms obtained for filaments (A) and 3D printed tablets (B) with paracetamol.

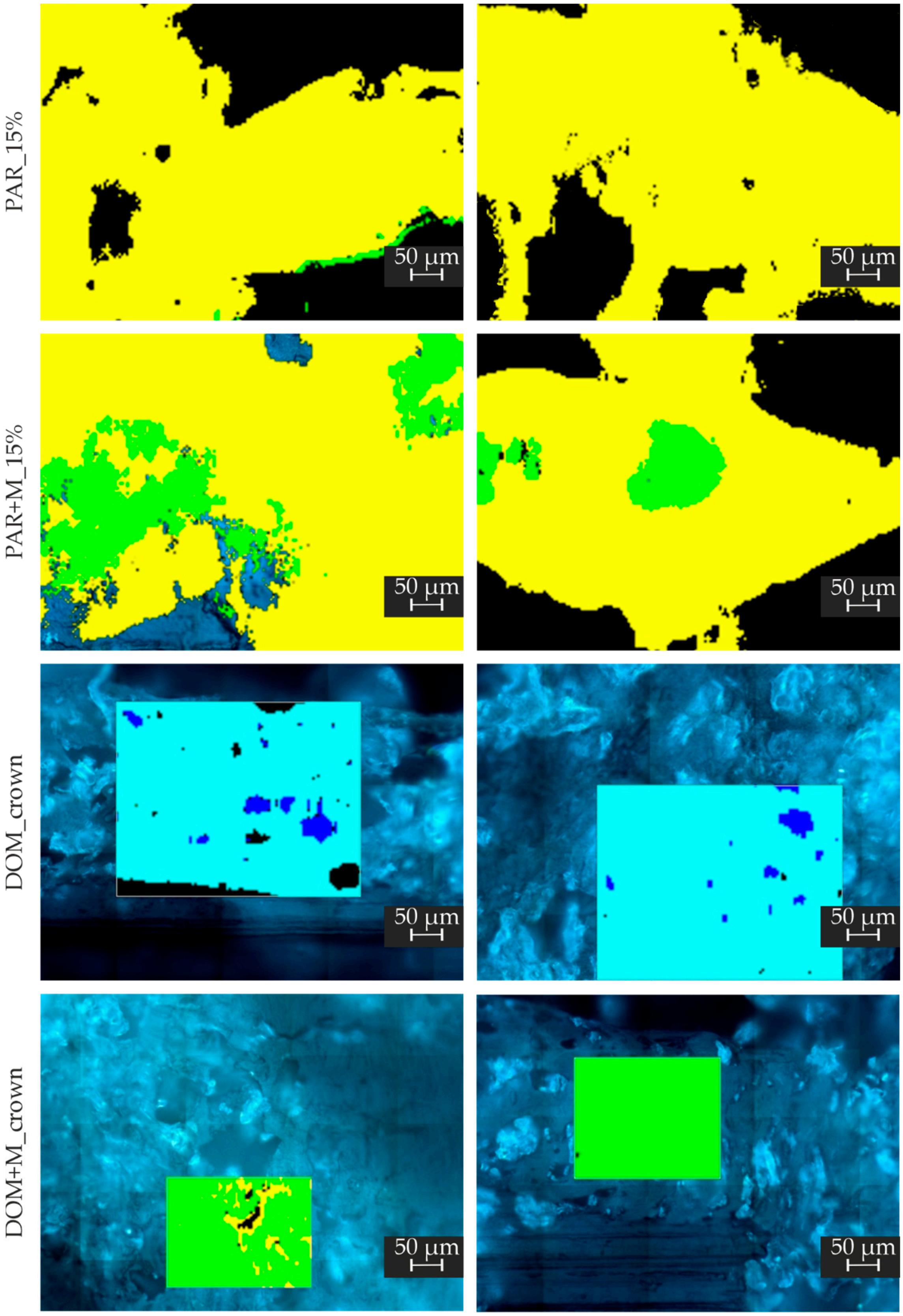

Figure S6 Raman maps of tablets made of filaments with different compositions with 50× magnification and 4 µm step used. Yellow is for APIs in amorphous form, green for crystalline, blue for crospovidone, cyan for PVA, and black for wax or unidentified spectra.
